# Effect of Nano-SiO_2_ Modification on Mechanical and Insulation Properties of Basalt Fiber Reinforced Composites

**DOI:** 10.3390/polym14163353

**Published:** 2022-08-17

**Authors:** Hechen Liu, Yu Sun, Yunfei Yu, Mingjia Zhang, Le Li, Long Ma

**Affiliations:** 1 Hebei Key Laboratory of Green and Efficient New Electrical Materials and Equipment, North China Electric Power University, Yonghua North Street No. 619, Baoding 071003, China; 2 State Key Laboratory of Alternate Electrical Power System with Renewable Energy Sources, North China Electric Power University, Beinong Road No. 2, Beijing 102206, China; 3 Key Laboratory of Physical and Chemical Analysis for Electric Power of Hainan Province, Hairui Road No. 23, Haikou 570100, China

**Keywords:** Nano-SiO_2_, basalt fiber, surface modification, insulation strength, compound materials

## Abstract

Basalt fiber (BF) has high mechanical strength, good insulation performance and low cost. It is suitable to be used as reinforcement material in the manufacture of electrical equipment. However, the large surface inertia of basalt fiber makes it difficult to combine with the matrix material, which seriously limits its service life and application scenarios. In addition, the serious vacancy in the research of insulation properties also limits its production and application in the electrical field. Therefore, in order to solve the problem of difficult bonding between basalt fiber and resin matrix and make up for the research blank of basalt fiber composites in insulation performance, this paper provides a basalt fiber modification method—SiO_2_ coating, and tests the insulation and mechanical properties of the modified composite. We used nano-SiO_2_ coating solution to modify basalt fiber, and manufactured BF/resin composite (BFRP) by hand lay-up and hot-pressing technology, and experimentally analyzed the influence of nano-SiO_2_ content on the mechanical and insulation properties of the modified composite. Fourier transform infrared spectrum and scanning electron microscope analysis showed that nano-SiO_2_ was successfully coated on basalt fibers. Through the microdroplet debonding test, it was found that the IFSS of fiber/resin was improved by 35.15%, 72.97 and 18.9%, respectively, after the modification of the coating solution with SiO_2_ concentration of 0.5%, 1% and 1.5%, showing better interface properties; the single fiber tensile test found that the tensile strength of the modified fiber increased slightly. Among all composites, 1 wt% SiO_2_ coating modified composites showed the best comprehensive properties. The surface flashover voltage and breakdown field strength reached 13.12 kV and 33 kV/mm, respectively, which were 34.6% and 83% higher than unmodified composite. The dielectric loss is reduced to 1.43%, which is 33.8% lower than the dielectric loss (2.16%) of the untreated composite, showing better insulation ability; the tensile strength, bending strength and interlaminar shear strength were increased to 618.22 MPa, 834.74 MPa and 16.29 MPa, respectively, which were increased by 53%, 42.4% and 59.7%, compared with untreated composites. DMA and glass transition temperature showed that the modified composite had better heat resistance. TGA experiments showed that the resin content of the modified composite increased, and the internal structure of the composite became denser.

## 1. Introduction

Fiber reinforced composites (FRP) have been widely used in many fields such as construction and machinery [1,2]. According to different reinforcement materials, FRP can be divided into glass fiber reinforced composites (GFRP), aramid fiber reinforced composites (AFRP), carbon fiber reinforced composites (CFRP), etc. Among them, basalt fiber reinforced composites have attracted more and more attention because of their excellent performance and low cost. In the realm of international materials, basalt fiber (BF) is a novel green environmental protection material. It is created from natural basalt through melting and wire drawing, and the production method will not result in wastewater, waste gas or waste residues, making it very environmentally friendly [3]. In addition to its excellent mechanical properties such as high strength (tensile strength of single wire can reach 2000–4840 MPa), high modulus (91–110 GPa), it also has the characteristics of high electrical insulation, high and low temperature resistance (−269–700 °C), acid and alkali aging resistance, low cost and so on. Therefore, it is suitable to be used as a reinforcing material in manufacturing insulating pull rods, composite cross arm mandrel materials, insulating barriers, insulating operating rods and other electrical insulation equipment [4,5,6]. However, in basalt fiber reinforced composites (BFRP), the BF surface is inert and the interface bonding strength with the matrix resin is low. The weak interface interaction will lead to the failure of the composite structure, thus limiting its service life and application. Therefore, it is imperative to modify the fiber surface to enhance the interface bonding strength between fiber and resin [7,8,9].

Numerous techniques are used to modify fibers, such as acid–base treatment [10], coupling agent treatment method [11], surface coating method [12] and plasma treatment method [13]. Among them, surface coating method is frequently employed because of its straightforward operation, positive modification effect and suitability for industrial production. At the same time, prior studies have demonstrated that the interfacial bonding strength between epoxy matrix and basalt fiber can be considerably increased by including nanoparticles into the coating to create a nano hybrid coating. Kim et al. modified MMT with silane, which improved the compatibility between montmorillonite and hydrophobic polymer, allowed more resin to be intercalated between fiber and MMT, reduced the number of voids, and thus improved the interfacial bonding strength [14]. γ-Methacryloyloxypropyl trimethoxysilane(KH570) and γ-Aminopropyl triethoxysilane (KH550) modified silicon carbon black nanoparticles were utilized by Gong’s team to examine the impact of these modifications on basalt fiber composites. The findings demonstrated a significant improvement in the bending and impact resistance of modified basalt fiber reinforced unsaturated polyester resin (BF/UPR) composites. It was also discovered that the modification effect of silicon carbon black particles treated with KH550 on BF/UPR composites was better, reaching 105.1 MPa and 80.0 kJ/m^2^, respectively, increasing by 61.2% and 20.3% [15]. Current research is primarily focused on the impact of nano particle modification on the mechanical properties of BFRP. In contrast, less study is being carried out on the impact of composite insulation qualities, which restricts the usage of BFRP in the electrical area.

An environmentally friendly substance with a composition similar to basalt fiber is nano-SiO_2_. It is widely utilized in coatings, rubber, plastics and other areas because of its rich content, high economy, large specific surface area, tiny particle size and strong surface adsorption. Pallaj et al. made composite coatings of micro-SiO_2_ and nano-SiO_2_ particles with titanium dioxide, talc powder and epoxy polyamide base, respectively, and coated them on the surface of sandblasted steel. The composite materials were aged in salt spray experiment. Research showed that the coating with nano-SiO_2_ had better corrosion resistance than that with micro-SiO_2_ [16]. Pham, P used nano-SiO_2_ to toughen epoxy resin. Compared with pure epoxy resin, when the content of Nano-SiO_2_ is 7 wt%, the fracture toughness of toughened epoxy resin was increased from 0.61 MPa to 0.71 MPa, an increase in 16.7%, and the glass transition temperature was increased from 140 °C to 146 °C, indicating that the addition of Nano-SiO_2_ significantly improved the toughness and high temperature resistance of epoxy resin [17]. Some scholars have used nano-SiO_2_ to treat the surface of fibers. Zhu et al. grafted a SiO_2_ coating with nano porous structure on the surface of PBO fibers by sol-gel method, using 3-glycidyloxypropyltrimethoxysilane (GPTMS) as silane coupling agent and tetraethyl orthosilicate (TEOS) as silicon source. It was found that the bonding strength between the modified PBO/SiO_2_ fiber and epoxy resin was significantly improved, the interfacial shear strength was increased by 114.8% and the hydrophobicity was also significantly enhanced [18]. Tang et al. manufactured aramid fiber insulating paper by treating aramid fiber with 1% SiO_2_. The aramid fiber insulating paper was subjected to mechanical and electrical tests both before and after modification. A strong interface effect between nano-SiO_2_ and aramid fiber was found to increase the tensile strength, elongation at break and AC breakdown strength of 1 wt% SiO_2_ treated aramid fiber insulating paper by about 7.5%, 10% and 14%, respectively, when compared to unmodified aramid fiber insulating paper. This improved the mechanical and insulating properties of the aramid fiber insulating paper [19]. However, due to the high surface free energy of nano-SiO_2_, it is prone to agglomeration, so it is necessary to modify its surface to avoid agglomeration.

In order to provide some data references for the application of BFRP in the field of electrical materials, nano-SiO_2_ modified by silane coupling agent and succinic anhydride was used to coat the surface of BF, and the insulation and mechanical properties of the modified composite were explored.

## 2. Experimental

### 2.1. Materials

The experimental raw materials used in this study are as follows: Basalt fiber plain fabric, the monofilament diameter is 13 ± 1 μm, area density 300 ± 15 g/m^2^, Sichuan Aerospace Tuoxin basalt Industry Co., Ltd., Chengdu, China; Bisphenol A epoxy resin DGEBA, epoxy value 0.51–0.54 eq/100 g, epoxy equivalent 184–195 g/eq, industrial pure, pelim Electric Technology Co., Ltd. (Quzhou, China); Methyl hexahydrophthalic anhydride MHHP, 2,4,6-tris (Dimethylaminomethyl) phenol DMP-30, purity ≥ 95%, Guangzhou Desheng Chemical Co., Ltd., Guangzhou, China. Nano-SiO_2_ (30 ± 5 nm, purity ≥ 99.5%), Shanghai McLean Biochemical Technology Co., Ltd., Shanghai, China; Dimethylformamide DMF, Shandong Mingcheng new materials Co., Ltd. (Binzhou, China); γ- Aminopropyl triethoxysilane KH550 (purity ≥ 99%), Jinan xingfeilong Chemical Co., Jinan, China, Acetone, purity ≥ 99.5%, Aldrich Trading Co., Ltd. (Shanghai, China); Succinic anhydride, purity ≥ 99.5%, Tianjin kemio Chemical Reagent Co., Ltd., Tianjin, China. Some properties of materials used in the experiment are shown in the Table 1.

### 2.2. Nano-SiO_2_ Modification

**Figure 1:**
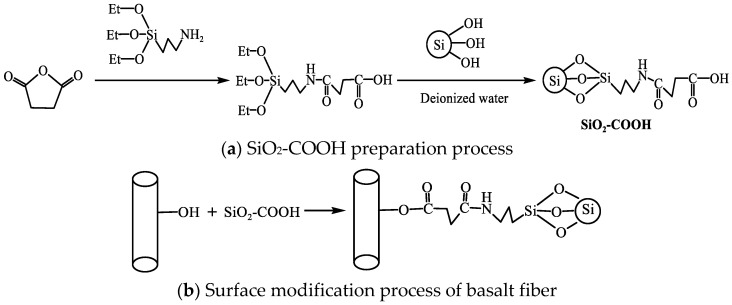
Principle of chemical reaction. (**a**) SiO_2_-COOH preparation process. (**b**) Surface modification process of basalt fiber.

In order to avoid agglomeration of nano-SiO_2_, silane coupling agent was used to modify it. Take the preparation of 0.1 mol carboxylated SiO_2_ (SiO_2_-COOH) as an example: First, about 0.1 mol of nano-SiO_2_ and 2 mol of deionized water was added to an appropriate amount of DMF, and ultrasonic dispersion was carried out for 0.5 h at 180 W output power to obtain mixture A. Then, we added 1 mol of KH550 and 1 mol of succinic anhydride in DMF, and mixed solution B was obtained by magnetic stirring at 80 °C for 3 h. The solution A and B were mixed and stirred at 40 °C for 3 h, and then centrifuged, washed with alcohol and dried to complete the surface carboxylation of Nano-SiO_2_. The reaction process is shown in Figure 1a.

### 2.3. Surface Modification of BFRP

In order to remove the chemical substances on the surface of basalt fiber fabric, continuous basalt fiber was extracted by Soxhlet extraction method. The fiber fabric should be placed in a Soxhlet extractor, extracted with acetone for 12 h, thoroughly washed with deionized water five times, and then placed in a vacuum drying oven to finish drying.

**Figure 2:**
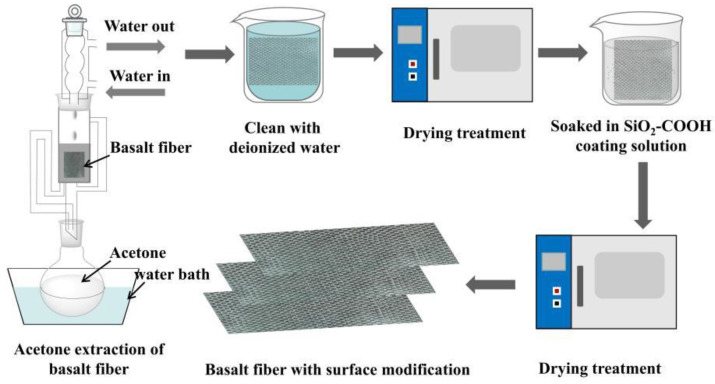
Experimental operation process.

The coating solution with the mass fraction of 0.5 wt%, 1 wt% and 1.5 wt% was made by equally dispersing SiO_2_-COOH in the mixed solution with the mass ratio of absolute ethanol to water of 80:20. We then soaked the extracted basalt fiber in the coating solution for 1 h and dried it in a drying oven at 50 °C for 3 h. The reaction is shown in Figure 1b, and the experimental operation process is summarized in Figure 2. The sample abbreviations and treatment methods required in the experiment are summarized in Table 2.

### 2.4. Preparation of BFRP

DGEBA, MHHPA and DMP-30 were blended at the mass ratio of 100:75:0.3. Then, stirred the solution with magnetic force for 10 min at room temperature to make it uniform, and put it into a vacuum drying oven for defoaming until there were no bubbles in the solution. Finally, the treated basalt fiber was soaked in the prepared epoxy resin solution, and the basalt fiber/epoxy resin composite was prepared by hand lay-up and hot-pressing technology.

### 2.5. Testing and Characterization

#### 2.5.1. Surface Morphology Test

The chemical composition changes of nano-SiO_2_ and SiO_2_-COOH were examined using Fourier transform infrared spectroscopy (FTIR) by sensor II (255) spectrometer of the Brooke company in Saarbruken, Germany (wave number scanning range: 400–4000 cm^−1^, resolution: 0.5 cm^−1^). This analysis was carried out using the method of making iodine bromide tablets.

The morphological alterations of the fiber surface were examined using the American Fei Company’s field emission scanning electron microscope (SEM) Nova nano-450. The surface of the fibers was sprayed with a small layer of gold before the experiment to increase the conductivity of the fiber. The acceleration voltage was 10 kV, and the sample was amplified to 1000–5000 times. The scales used are 1:2000 and 1:1100, respectively.

YG163 of Jigao Testing Instrument Co., Ltd., Wenzhou, China was used to test the micro debonding and monofilament strength of single basalt fiber before and after modification. Single-fiber tensile test was carried out at 2 mm/min tensile rate. When measuring the interfacial shear strength (IFSS) of fibers, in order to prevent the fibers from being broken during peeling, the size of microspheres should be controlled to avoid excessive embedding length.

#### 2.5.2. Insulation Performance Test

For the breakdown strength test, reference GB/T 16927.1-2011, used parallel plate electrodes to boost the sample at a uniform speed of 2 kV/s until the sample was broken down.

Dielectric loss test reference GB/T 1409-2006, applied 4 kV voltage to the sample with Agilent-4294A impedance analyzer.

Surface flashover test reference GB/T 1406.1-2006, finger electrodes (the distance between electrodes was 8 mm) were used to boost voltage at a uniform speed of 2 kV/s until the surface of the sample was broken down.

#### 2.5.3. Physical and Mechanical Test

The 100ST universal mechanical testing machine of Tennessee Orson was used to conduct mechanical tests on the samples. Five samples were tested in each group, and the final experimental results were averaged. We conducted bending tests in accordance with ASTM D790, using the three-point bending method, at a test speed of 2 mm/min; conducted tensile test according to ASTM D638, and the test speed was 10 mm/min; performed an ASTM 3846-compliant interlaminar shear test on the sample, measured its interlaminar shear strength (ILSS) and performed a SEM test on the sample’s cross-section to check for fiber and resin separation.

The water absorption tests were conducted by immersing the sample in deionized water at 23 °C and 100 °C for 24 h according to ASTM D570.

The dynamic thermomechanical analysis (DMA) experiment was carried out on the sample. Using the single cantilever test mode, the room temperature increased from room temperature to 250 °C at a constant speed of 5 °C/min, with a frequency of 2 Hz and an amplitude of 10 μm.

TGA 4000 of PerkinElmer Co., Ltd. (Waltham, Massachusetts, America) was used to carry out hermos-gravimetric analysis (TGA) experiment on the samples, and the changes in fiber content in the composites before and after modification were analyzed. The experimental temperature was 35–800 °C, and the heating rate was 10 °C/min.

## 3. Results and Discussion

### 3.1. Surface Morphology and Mechanical Strength of BF

#### 3.1.1. FTIR

**Figure 3:**
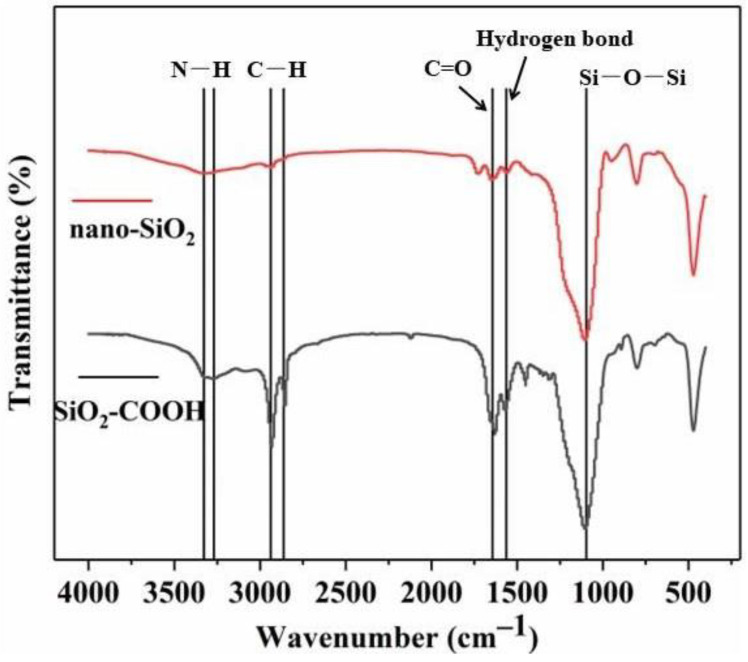
Infrared spectra of nano-SiO_2_ and SiO_2_-COOH.

As can be seen from Figure 3, compared with nano-SiO_2_, the infrared spectral image of SiO_2_-COOH changes significantly. Wavenumbers at 3280 and 3325 cm^−1^ have new absorption peaks, which belong to the symmetric and asymmetric vibration of -N-H bond [20]. The new absorption peak at 2856–2959 cm^−1^ is attributed to the tensile vibration of -C-H bond, which also shows that the surface of silicon dioxide is composed of organic groups; The transmittance at 1640 cm^−1^ is significantly reduced, which belongs to the tensile vibration of C=O in esters [21], and is the performance of KH550 grafted onto SiO_2_; The infrared wave at 1560 cm^−1^ is the deformation mode of hydrogen bond between -N-H and -O-H, and the tensile vibration peak of Si-O-Si is near 984 cm^−1^; the above phenomena show that KH550 has been successfully grafted on nano-SiO_2_ and the surface modification is successful.

#### 3.1.2. SEM Experiment

**Figure 4:**
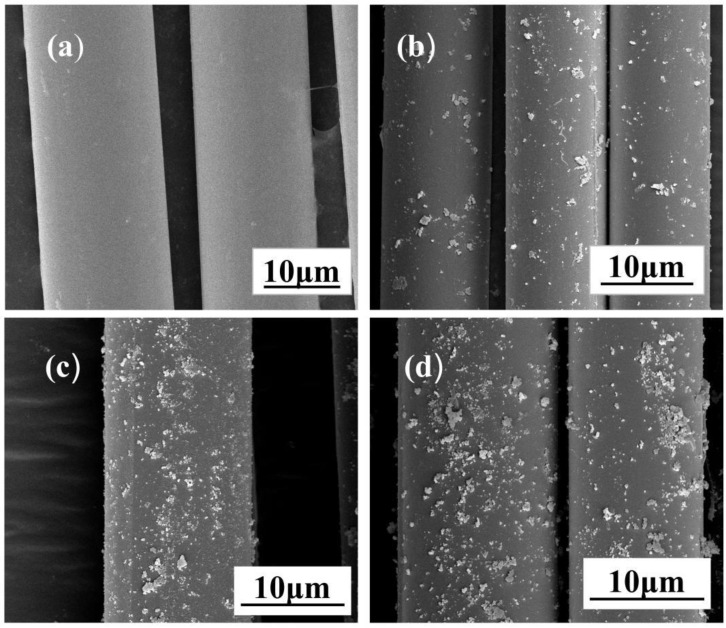
SEM images of different BF surfaces: (**a**) ABF, (**b**) 0.5-SiO_2_-BF, (**c**)1-SiO_2_-BF, (**d**) 1.5-SiO_2_-BF.

Figure 4a depicts the surface morphology of ABF, which demonstrates that the fiber surface is extremely smooth. Surface roughness increased to some extent in BF treated with 0.5 wt% SiO_2_-COOH, but the coating was uneven, as shown in Figure 4b; Figure 4c shows the electron microscope image of 1-SiO_2_-BF. SiO_2_-COOH particles are evenly distributed on the fiber surface, which greatly improves the roughness of the fiber surface; Figure 4d depicts the fiber image modified by 1.5 wt% SiO_2_-COOH. It can be seen that some nanoparticles are evenly dispersed on the fiber surface, while some SiO_2_-COOH molecules attract each other to form aggregates on the fiber surface due to their close proximity. In order to further measure the coating efficiency of different concentrations of SiO_2_-COOH coating solution, the quality of clean fibers before and after SiO_2_-COOH treatment was tested. The results showed that the content of SiO_2_-COOH adhered to the fiber surface increased with the increase in coating solution concentration. The specific results are shown in Table 3.

#### 3.1.3. IFSS

**Figure 5:**
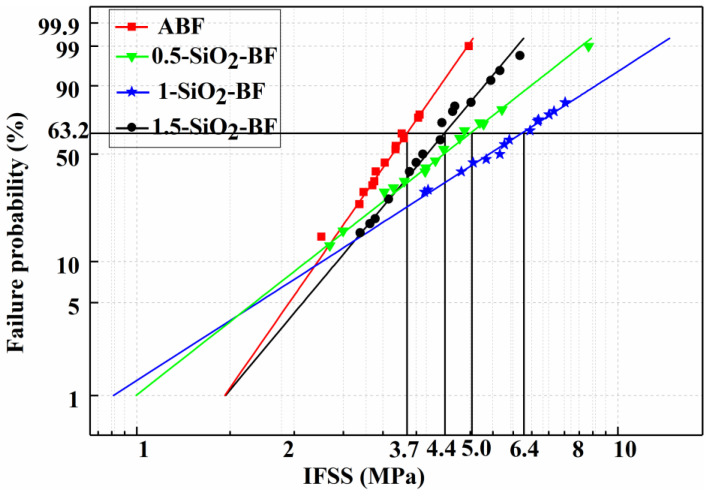
IFSS of fiber/resin before and after modification.

The interfacial bonding strength between single fiber and resin was tested by microdroplet debonding test. The Weibull distribution of IFSS of BF/resin treated with various concentrations is shown in the Figure 5. The experiments show that the IFSS of BFs treated with SiO_2_-COOH increases in varying degrees. Compared with IFSS of ABF, IFSS of basalt fiber modified by 0.5, 1 and 1.5 wt% SiO_2_-COOH coating reached 5, 6.4 and 4.4 MPa, increased by 35.15%, 72.97% and 18.9%, respectively. These results can be attributed to the fact that SiO_2_-COOH particles increase the roughness of the fiber surface, thereby enhancing the interfacial adhesion between BF and the matrix resin [22]. However, the increase in IFSS is closely related to the concentration of SiO_2_-COOH coating solution. Low concentration (0.5 wt%) coating solution will lead to uneven coating on the fiber surface and poor modification effect. High concentration of SiO_2_-COOH coating solution (1.5 wt%) may form agglomeration on the fiber surface, limiting the combination of fiber and resin. BFRP modified with 1 wt% SiO_2_-COOH coating showed the best interfacial strength. Anyway, in general, using SiO_2_-COOH solution with appropriate concentration to coat the fiber surface is an effective way to improve its surface properties.

#### 3.1.4. Tensile Strength of Single-Fiber

**Figure 6:**
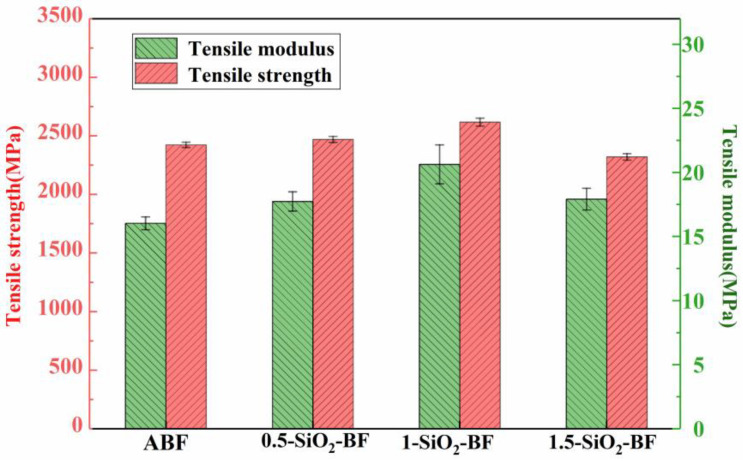
Tensile strength of fiber before and after modification.

The Figure 6 shows that the presence of SiO_2_-COOH particles changes the fiber’s tensile strength and elastic modulus to some amount, but the change is very minimal. The specific data are listed in Table 3. The tensile strength and elastic modulus of BF coated with 0.5 wt% and 1 wt% SiO_2_-COOH coating solution increased slightly, which may be because SiO_2_-COOH particles repaired the defects on the surface of the fiber, so that the stress was evenly transmitted to the interior of the fiber, thereby improving the tensile strength and elastic modulus of the fiber [23]. However, the tensile strength of BF coated with 1.5 wt% SiO_2_-COOH coating solution decreased, which may be due to the fact that SiO_2_-COOH particles gathered on the fiber surface and formed many defects, such as cracks. When tensile load was applied to the fiber, the cracks gradually expanded and extended from the coating/fiber interface to the fiber surface, thus forming many stress concentration points on the fiber surface, resulting in uneven stress distribution on the fiber surface and accelerating the fracture of the fiber.

### 3.2. Insulation Performance of BFRP

#### 3.2.1. Dielectric Loss

**Figure 7:**
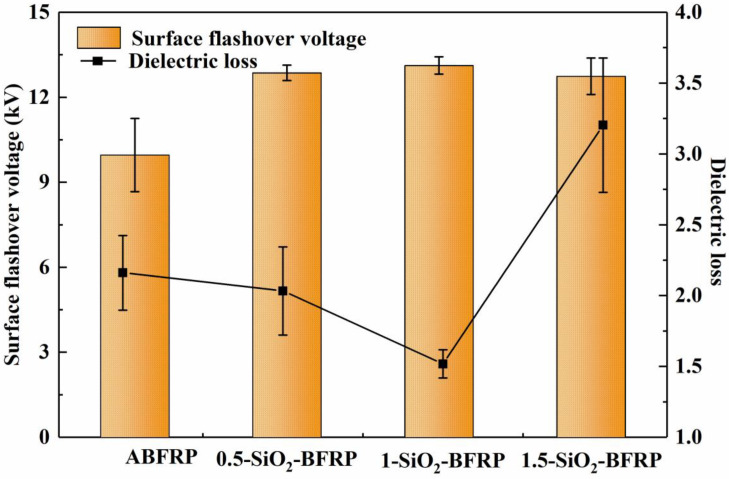
Dielectric loss and surface flashover voltage of composites treated with different mass fraction of SiO_2_-COOH.

Excessive dielectric loss (tanδ) of insulation materials will lead to the accumulation of internal heat and accelerate the thermal aging of composite materials. Therefore, reducing the tanδ of composite materials is essential to improve its insulation life. Composite tanδ is primarily caused by conductivity and polarization loss [24]. Figure 7 illustrates how the tanδ first reduces and then grows as the mass fraction of SiO_2_-COOH increases. Tanδ for 1-SiO_2_-BFRP is at its lowest (1.43%), 33.8% less than for ABFRP (2.16%). There are two reasons why the dielectric loss of 1-SiO_2_-BFRP sample is significantly lower than that of ABFRP sample. One of them is that SiO_2_-COOH particles increase the fiber surface’s roughness, which enhances the mechanical meshing effect between the fiber and resin, makes the interface more closely combined. The tightly coupled interface effectively prevents carriers from moving through the polymer, restricts the production and growth of leakage current and so lowers conductivity loss. Second, SiO_2_-COOH particles on the fiber surface in 1-SiO_2_-BFRP will join the resin to create hydrogen bonds that attach the resin polymer chain to the fiber surface, preventing the resin long chain from becoming polarized and so minimizing polarization loss. The tanδ of 1-SiO_2_-BFRP sample decreased significantly with the reduction in conductivity loss and polarization loss. However, the dielectric loss of 0.5-SiO_2_-BFRP sample is not much lower than that of ABFRP. The reason is that the SiO_2_-COOH concentration is too low, so that there are too few SiO_2_-COOH particles on the fiber surface, and the fiber modification effect is poor. As a result, the conductivity loss and polarization loss are not significantly reduced, and the dielectric loss is not much reduced. However, the dielectric loss of 1.5-SiO_2_-BFRP sample is significantly higher than that of 1-SiO_2_-BFRP. The reason is that when the concentration of SiO_2_-COOH is too high, a large number of nanoparticles form large-scale aggregates on the surface of the fiber, and the aggregates interact with each other to form conductive paths; aggregates also make it harder for fibers and resins to be mixed closely, generating a lot of flaws at the composites’ interface that make it simpler for conductive channels to emerge. Because carriers can easily be transmitted over these conductive channels, conductivity loss is increased.

#### 3.2.2. Surface Flashover Voltage

Figure 7 shows that SiO_2_-COOH coated composites have greater flashover voltages than ABFRP. The 1-SiO_2_-BFRP samples have the highest average surface flashover voltage, 13.12 kV, which is 34.6% greater than that of ABFRP (9.75 kV). When the concentration of SiO_2_-COOH continues to increase, the surface flashover voltage decreases slightly. The rationale is that by coating nanoparticles on the fiber surface, many deep traps will be introduced into the composite, allowing them to collect electrons and inhibit secondary electron emission on the surface, which will ultimately enhance the flashover voltage [25,26]. When the concentration of nano materials is too high to form aggregates, the adsorption capacity of aggregates to carriers is weakened, resulting in the increase in carrier mobility and the decrease in surface flashover voltage [27].

#### 3.2.3. Breakdown Strength

**Figure 8:**
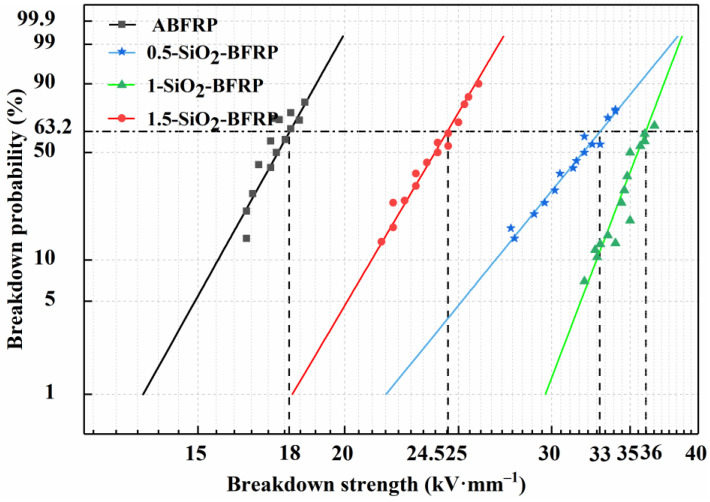
Breakdown strength of composites treated with different mass fraction of SiO_2_-COOH.

Figure 8 displays the four composites’ AC voltage breakdown strengths. The breakdown field strength of the composites is the highest at 33 kV/mm when the concentration of SiO_2_-COOH is 1 wt%, which is much higher than that of the ABFRP, a rise of 83%. The reason is that fibers are uniformly grafted with an adequate quantity of SiO_2_-COOH, which makes the resin more closely bound nearby and greatly lowers the quantity of pores and cracks in the composite. When voltage is applied, the internal electric field of the composite is more uniform, which inhibits the production of partial discharge. In addition, the existence of nanoparticles also limits the occurrence of electron avalanche [28,29], extending the breakdown time of the composite. While a SiO_2_-COOH coating solution containing 1.5 wt% will aggregate on the fiber surface, this reduces the area where the fiber and resin can bond, decreases the fiber’s wettability, introduces numerous pores and defects at the interface, and is vulnerable to partial discharge, all of which reduce the breakdown strength of the composite.

### 3.3. Physical and Mechanical Properties of BFRP

#### 3.3.1. Water Absorption

**Figure 9:**
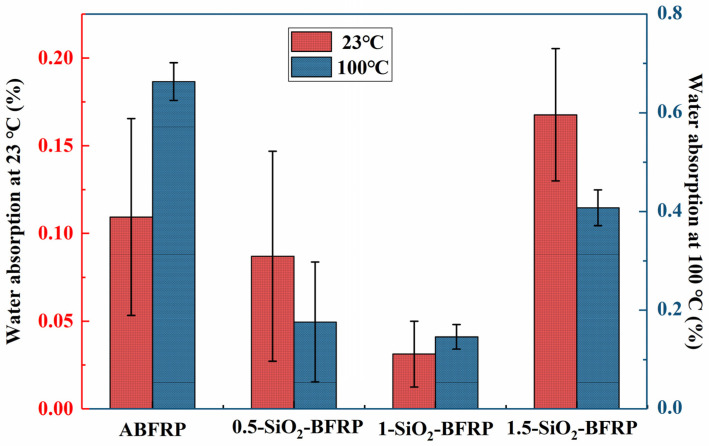
Water absorption of composites treated with different mass fraction of SiO_2_-COOH.

The water absorption of composites is related to many factors such as pores and impurities at the interface of composites, among which pores are the decisive factor affecting water absorption. Figure 9 shows that all samples’ water absorption at 100 °C is significantly higher than at 23 °C because high temperatures are more damaging to the composite material’s interface, making it simpler for water molecules to enter the interior of the material and cause cracks and water channels on the interface. In the two groups of experiments, the water absorption of 1-SiO_2_-BFRP is the smallest, because at this time, the SiO_2_-COOH particles are most evenly distributed on the fiber [30], and the combination degree of fiber and resin is the highest, resulting in the lowest porosity on the composite interface, so the water absorption is the lowest. The water absorption of 1.5-SiO_2_-BFRP is significantly higher than that of 1-SiO_2_-BFRP, because the aggregates accumulated on the fiber surface, which make the fiber less wettable and increase the number of pores at the interface, and thus makes it easier for water molecules to bind to the surface and absorb water molecules. Figure 6 shows that the experimental errors for abfrp, 0.5-SiO_2_-BFRP, and 1.5-SiO_2_-BFRP are considerable. The treatment effect of 0.5 wt% and 1.5 wt% concentration SiO_2_-COOH solution on the fiber surface may be uneven, which causes the interface bonding strength of some areas of the composite to be high, with few pores and low water absorption; in other areas of the composite, the interface bonding strength is low, with many pores and high water absorption. The final data acquired are extensively dispersed, with a large experimental error value. While the fiber modified with 1% SiO_2_-COOH solution uniformly boosts the composite’s interface strength, the number of holes is reduced, and the water absorption is significantly reduced. As a result, the experimental error is minimal, and the results are not dispersive. In terms of water absorption and inaccuracy, 1-SiO_2_-BFRP performs the best overall, which is consistent with the previous experimental results.

#### 3.3.2. Mechanical Property of BFRP

##### Tensile Strength

Table 4 shows that the tensile strength of 1-SiO_2_-BFRP is 618.22 MPa, which is 53% higher than that of ABFRP (404.12 MPa). It demonstrates that coating SiO_2_-COOH on the surface of the fiber can considerably increase the composite’s tensile capabilities, since SiO_2_-COOH is cemented in the matrix resin as a rivet, strengthening the interface bonding ability between the fiber and the resin, debonding points decrease as a result [31,32]. When a tensile load is given to the composite, the improved interface may evenly transfer the stress to the fiber, minimizing stress concentration, hence improving the composite’s tensile strength [33]. However, if the coating solution concentration is too low (0.5 wt%), SiO_2_-COOH cannot be uniformly coated on the fiber surface, resulting in poor interfacial bonding. Excess particles on the fiber surface form aggregates when the concentration of SiO_2_-COOH solution is too high. These large-scale agglomerations will cause stress concentration in the composite, expedite composite failure and diminish tensile strength.

##### Bending Strength

As shown in Table 4, the bending properties of the composites modified with different concentrations of SiO_2_-COOH have been improved to a certain extent, among which the bending strength of 1-SiO_2_-BFRP has the greatest improvement, which is 248 MPa (42.4%) higher than that of ABFRP. SiO_2_-COOH is connected to the fiber surface, increasing its roughness and making it simpler for the resin to adhere to the fiber; at the same time, by introducing carboxyl groups and other active functional groups on the surface of the fiber, the wettability of the fiber is improved, so as to improve the interfacial bonding strength of the composite. By allowing the load energy to be distributed evenly between the resin and the fiber and allowing some of the energy to dissipate during the plastic deformation of the resin, a good interface structure reduces the formation and growth of microcracks and increases the bending strength of the composite [34]. In 1.5-SiO_2_-BFRP, the decrease in bending strength is due to the aggregation of a large number of aggregates on the fiber surface, which reduces the riveting effect of SiO_2_-COOH, reduces the wettability of the fiber, increases interface defects and leads to stress concentration.

##### Flexural Modulus of Elasticity

**Figure 10:**
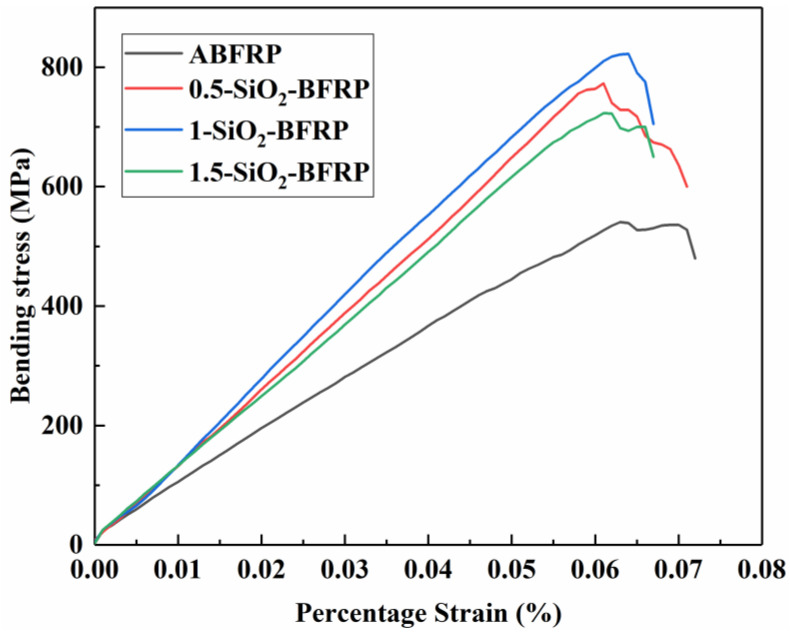
Stress–strain curves of composites treated with different mass fraction of SiO_2_-COOH.

Figure 10 shows that the SiO_2_-COOH modification dramatically alters the composite’s stress–strain curve and significantly increases the maximum bending stress and strain that the composite can withstand. The basalt fiber in the composite that has been treated with SiO_2_-COOH has a good interface and can withstand enough load to increase the composite’s strength [35,36]. At the same time, the stress–strain curve of SiO_2_-COOH modified composite broke instantaneously under the limit value of bending strength without further deformation, which is the damage caused by fiber fracture under limit load. Therefore, the surface modification of SiO_2_-COOH on the fiber can significantly improve the load capacity of the composite and make it have higher failure strength.

##### Interlaminar Shear Strength

**Figure 11:**
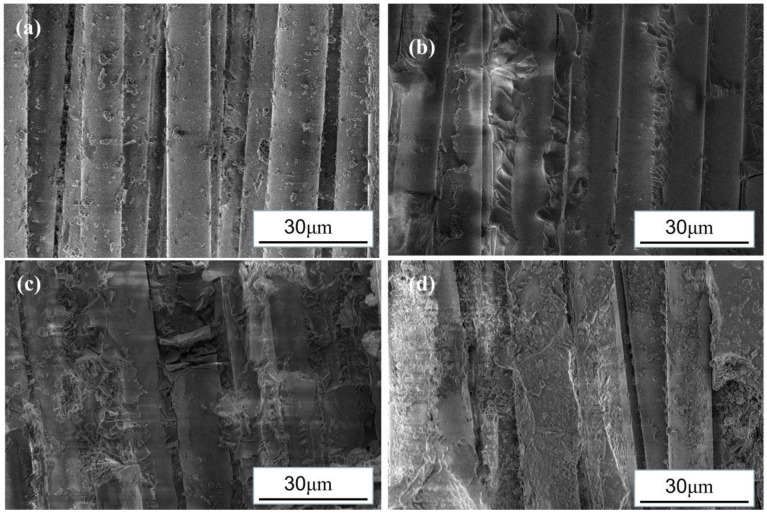
SEM images of shear fracture surface of composites treated with different mass fraction of SiO_2_-COOH. (**a**) ABFRP, (**b**) 0.5-SiO_2_-BFRP, (**c**)1-SiO_2_-BFRP, (**d**) 1.5-SiO_2_-BFRP.

The interlaminar shear strength (ILSS) of the modified composite is noticeably higher than that of the unmodified composite, as shown in Table 4. However, the precise modification effect is highly correlated with the concentration of SiO_2_-COOH. In order to further explain the influence of composite interface on its interlaminar shear properties, the samples after the test were tested by SEM. Figure 11 shows the results of SEM of the shear fracture surface. Figure 11a depicts the shear fracture surface of ABFRP. It is clear that the fiber surface is smooth and that only a small amount of resin has been adhered. The cause is poor compatibility between the resin and fiber. Interlayer collision causes the resin to peel off, revealing the entire fiber. It can be seen from Figure 11b that the content of coating resin on the fiber surface increases after 0.5 wt% SiO_2_-COOH surface modification. The best modification effect is reached when the concentration of SiO_2_-COOH is 1 wt%, as shown in Figure 11c. At this time, the surface of BF fiber is mostly covered by epoxy resin, and a large number of feather comb fracture marks appear at the connection between BF and resin. The above morphology represents that the resin has shear yield. SiO_2_-COOH particles act as mechanical dampers when a material is stressed, reducing slip and friction between fiber and resin to increase the composite’s shear resistance [37,38]; Figure 11d depicts the shear fracture surface of 1.5-SiO_2_-BFRP. It is clear that some fiber surfaces have resin that attaches strongly, whereas other fiber surfaces have resin that totally debonds. This is because the uniformly dispersed SiO_2_-COOH particles enhance the interfacial bonding strength of fiber/resin, while the aggregates formed by SiO_2_-COOH reduce the bonding strength of fiber and resin, resulting in a large difference in the interfacial bonding strength between fiber/resin [39].

##### DMA

**Figure 12:**
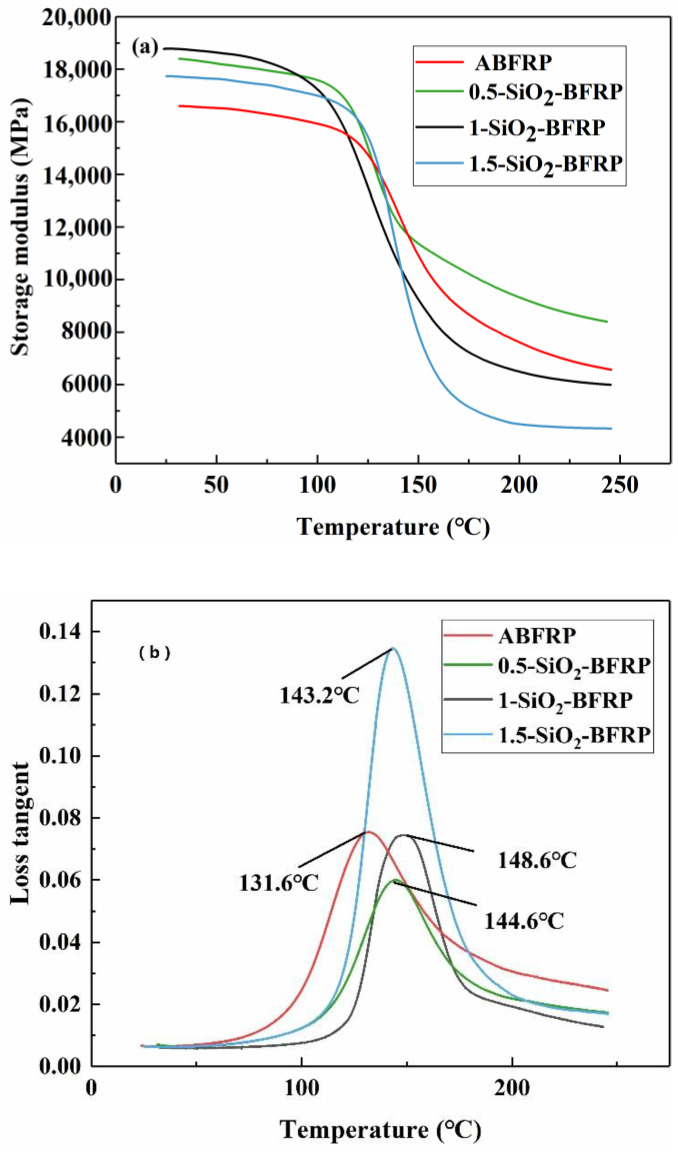
DMA experimental results of composites treated with SiO_2_–COOH at different concentrations: (**a**) Storage modulus–temperature curve; (**b**) loss tangent–temperature curve.

Figure 12 compares the composite’s DMA curve before and after the addition of SiO_2_-COOH. The storage modulus curves of the composites with temperature following four treatment procedures are shown in Figure 12a. At 30 °C, 1-SiO_2_-BFRP has the greatest storage modulus (1.13 times that of ABFRP). This is due to the composite’s greatly increased interfacial compatibility, which restricts the mobility of the polymer chain and causes the interface between the fiber and the matrix to transmit more stress, increasing the composite’s stiffness [40]. The worse the interfacial adhesion, the more energy dissipated by friction between matrix and fiber, and the larger the loss coefficient [41].

The value of loss tangent (tanδ) is equal to the ratio of loss modulus to storage modulus, which is also a very important parameter when studying the mechanical properties of fiber reinforced materials. The tanδ curves of the composites are shown in Figure 12b before and after modification. The temperature change curves in the picture show that the improved composite’s glass transition temperature (Tg) is moving overall in the direction of ascent. In comparison to the unmodified composite, the Tg of the composite rises by 17 °C to 148.6 °C at a SiO_2_-COOH concentration of 1 wt%. The polymer chain travels with greater internal friction resistance as a result of the molecular chain segment changing from a glass state to a rubber state as the temperature rises, leading to higher heat loss, poorer storage modulus and higher loss [42]. The interface of 1-SiO_2_-BFRP composite has high bonding strength, strong ability to restrict the movement of epoxy long chain and less friction energy consumption. Therefore, its storage modulus decreases relatively slowly, and the peak value of tanδ is low. When the concentration of SiO_2_-COOH is too high (1.5 wt%), the agglomerations on the fiber surface hinder the combination of resin and fiber, and the interfacial adhesion is low, resulting in higher energy dissipation.

##### TGA

**Figure 13:**
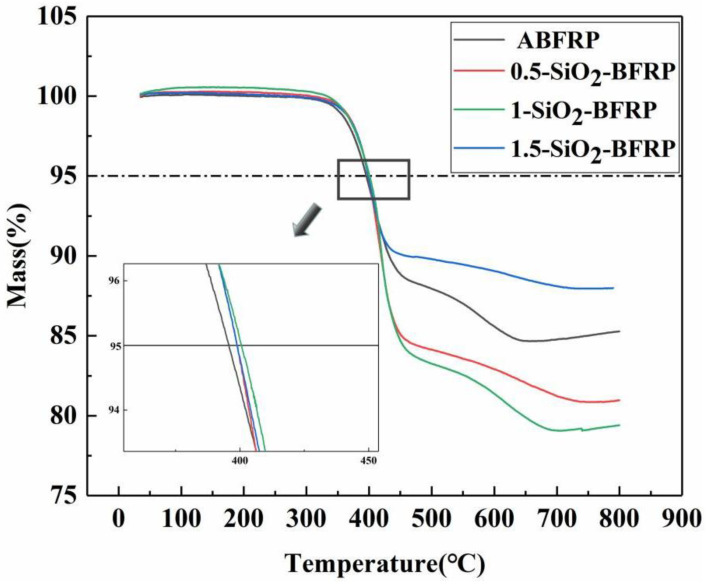
TGA experimental results of composites treated with SiO_2_-COOH at different concentrations.

The thermal stability of composites is usually evaluated by TGA. As shown in Figure 13, the decomposition of materials can be divided into two stages: decomposition of water and impurities (35–400 °C), resin carbonization and degradation process (400–800 °C). The mass reduction caused by the decomposition of water and impurities accounts for about 5% of the total mass, so the temperature corresponding to 95% of the initial mass is called the initial decomposition temperature [7]. Since the decomposition temperature of basalt fiber and SiO_2_ is much higher than 800 °C, the reduction in material quality after 400 °C is completely attributed to the carbonization and degradation of resin. Figure 13 shows that the initial decomposition temperature of the composite modified by SiO_2_-COOH is roughly the same, at about 400 °C, which is a little higher than the initial decomposition temperature of ABFRP (395 °C). This may be because the fiber and resin have a stronger bond, which decreases the resin’s fluidity and raises the composite’s thermal stability. Moreover, at 800 °C, the residual mass of the composite changes significantly. The residual mass of ABFRP, 0.5-SiO_2_-BFRP, 1-SiO_2_-BFRP and 1.5-SiO_2_-BFRP are 85%, 88%, 81% and 79%, respectively. Because the content of SiO_2_ is too low to be ignored, we believe that most of the residual part belongs to basalt fiber. It can be seen that the basalt fiber content in 0.5-SiO_2_-BFRP and 1-SiO_2_-BFRP in the composites is lower than that in ABFRP. The reason may be that the SiO_2_ treated composites reduce the interface defects, the dense structure between the fiber and the resin makes the resin completely infiltrate the fiber, the resin content is relatively increased, and the fiber content is relatively reduced; the basalt fiber content in 1.5-SiO_2_-BFRP, which may be due to the agglomeration on the fiber surface, which prevents the effective combination of fiber and resin, resulting in many defects and air gaps in the composite, so that the resin content is relatively reduced and the fiber content is relatively increased.

## 4. Conclusions

In this experiment, nano-SiO_2_ was modified with silane coupling agent KH550 to produce SiO_2_-COOH. Layer solutions with concentrations of 0.5 wt%, 1 wt% and 1.5 wt% were prepared with SiO_2_-COOH to modify BFRP. Finally, a series of insulation and mechanical tests were carried out on the composites before and after modification. It was found that the modification effect of SiO_2_-COOH coating solution was closely related to its concentration. Among all the experimental samples, the coating solution prepared by 1 wt% SiO_2_-COOH had the best modification effect on the composites, and 1-SiO_2_-BFRP showed the best interface strength and overall performance. The specific experimental results were as follows:

(1) The microdroplet debonding test and tensile test were carried out on single fibers. The results showed that the IFSS of BFs modified by coating increased to a certain extent, among which the increase in 1-SiO2-BF was the highest, which was 72.97 % higher than that of ABF. The tensile strength and elastic modulus of 0.5-SiO2-BF and 1-SiO2-BF increased to a certain extent, while the tensile strength of 1.5-SiO2-BF decreased slightly compared with ABF.

(2) Compared with the unmodified ABFRP, the electrical characteristics of the modified 1-SiO_2_-BFRP were significantly improved: the dielectric loss was reduced by 33.8%, the surface flashover voltage was increased by 34.6% and the breakdown field strength was increased by 83%.

(3) Mechanical experiments on the composites showed that the mechanical properties of the composites after surface treatment were significantly improved. Compared with ABFRP, the tensile strength of 1-SiO_2_-BFRP increased by 53%, the bending strength increased by 42.4% and the interlaminar shear strength increased by 59.7%. DMA experiments showed that the storage modulus of 1-SiO_2_-BFRP was 13% higher than that of ABFRP, and the glass transition temperature also moved to a higher direction, which was 17 °C higher than that of ABFRP, reaching 148.6 °C. This shows that the composite showed better interfacial bonding strength and heat resistance.

(4) TGA experiment showed that the fiber content in 0.5-SiO_2_-BFRP and 1-SiO_2_-BFRP were lower than that in ABFRP. Because the fiber/resin interface is closely bonded, the internal defects of the composite are less, and the relative content of the resin matrix is higher. The fiber content in the composite 1.5-SiO_2_-BFRP increased. This is due to the agglomeration of SiO_2_ particles, which hinders the combination of fiber and resin, resulting in pores and defects in the composites, so that the content of matrix resin is relatively reduced.

In conclusion, this study provides an effective way for surface modification of BF. After nano-SiO_2_ modification, the insulation and mechanical properties of BF composites are significantly improved, which is suitable for manufacturing electrical equipment with high insulation and high mechanical strength requirements.

## Figures and Tables

**Table 1:** Physical and chemical properties of experimental materials.

Materials	Structural Formula	Molecular Weight	Density (g/cm³)	Boiling Point (°C)
KH550	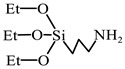	221.4	0.946	217
Succinic anhydride	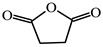	100.073	1.4	261
Nano-SiO_2_	O=Si=O	60.08	2.319–2.653	–
MHHPA	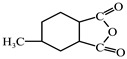	168.19	1.16	216
DMP-30	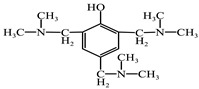	265.40	0.97–0.99	353.5
DGEBA	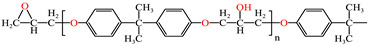	3100–7000	1.16	250–252
Acetone	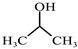	58.08	0.7899	56.5
Basalt fiber plain fabric	–	–	2.6–3.05	–

**Table 2:** Sample abbreviations and treatment methods.

Sample	Surface Treatment Method
ABF	Basalt fiber extracted with acetone
0.5-SiO_2_-BF	Basalt fiber modified by 0.5 wt% SiO_2_-COOH coating
1-SiO_2_-BF	Basalt fiber modified by 1 wt% SiO_2_-COOH coating
1.5-SiO_2_-BF	Basalt fiber modified by 1.5 wt% SiO_2_-COOH coating
ABFRP	Composites made of uncoated fibers
0.5-SiO_2_-BFRP	Composites made of 0.5 wt% SiO_2_-COOH coated fibers
1-SiO_2_-BFRP	Composites made of 1 wt% SiO_2_-COOH coated fibers
1.5-SiO_2_-BFRP	Composites made of 1.5 wt% SiO_2_-COOH coated fibers

**Table 3:** Adhesion efficiency of SiO_2_-COOH on fiber surface.

Samples	ABF	0.5-SiO_2_-BF	1-SiO_2_-BF	1.5-SiO_2_-BF
SiO_2_-COOH content covered on the fiber (wt%)	–	1.2 ± 0.25	1.9 ± 0.17	2.75 ± 0.23
Tensile strength of single fiber (MPa)	2423.9	2470.0	2617.7	2321.2
Elastic modulus of single fiber (MPa)	16.0	17.7	20.6	17.9

**Table 4:** Mechanical strength of composites before and after modification.

Samples	Tensile Strength (MPa)	Bending Strength (MPa)	Elasticity Modulus (MPa)	Interlaminar Shear Strength (MPa)
ABFRP	404.12	586.40	12,451.30	8.82
0.5-SiO_2_-BFRP	543.42	783.74	18,557.16	14.10
1-SiO_2_-BFRP	618.22	834.74	18,413.98	16.29
1.5-SiO_2_-BFRP	493.23	779.59	16,758.11	13.41

## Data Availability

The original data needed to reproduce these discoveries cannot be shared, because these data are also part of the ongoing research. The original data used to support the results of this study can be obtained from the communication author.
